# Characterization of Porous Cementitious Materials Using Microscopic Image Processing and X-ray CT Analysis

**DOI:** 10.3390/ma13143105

**Published:** 2020-07-12

**Authors:** Jinyoung Yoon, Hyunjun Kim, Sung-Han Sim, Sukhoon Pyo

**Affiliations:** 1 Department of Civil and Environmental Engineering, The Pennsylvania State University, State College, PA 16801, USA; jpy5278@psu.edu; 2 School of Civil, Architectural Engineering and Landscape Architecture, Sungkyunkwan University, Suwon 16419, Korea; hyunjun@skku.edu (H.K.); ssim@skku.edu (S.-H.S.); 3 School of Urban and Environmental Engineering, Ulsan National Institute of Science and Technology (UNIST), Ulsan 44919, Korea

**Keywords:** microscopic image processing, X-ray CT analysis, porous cementitious materials, 3D tomographic image

## Abstract

The use of lightweight concrete has continuously increased because it has a primary benefit of reducing dead load in a concrete infrastructure. Various properties of lightweight concrete, such as compressive strength, elastic modulus, sound absorption performance, and thermal insulation, are highly related to its pore characteristics. Consequently, the identification of the characteristics of its pores is an important task. This study performs a comparative analysis for characterizing the pores in cementitious materials using three different testing methods: a water absorption test, microscopic image processing, and X-ray computed tomography (X-ray CT) analysis. For all 12 porous cementitious materials, conventional water absorption test was conducted to obtain their water permeable porosities. Using the microscopic image processing method, various characteristics of pores were identified in terms of the 2D pore ratio (i.e., ratio of pore area to total surface area), the pore size, and the number of pores in the cross-sectional area. The 3D tomographic image-based X-ray CT analysis was conducted for the selected samples to show the 3D pore ratio (i.e., ratio of pore volume to total volume), the pore size, the spatial distribution of pores along the height direction of specimen, and open and closed pores. Based on the experimental results, the relationships of oven-dried density with these porosities were identified. Research findings revealed that the complementary use of these testing methods is beneficial for analyzing the characteristics of pores in cementitious materials.

## 1. Introduction

In recent years, lightweight cementitious materials have extensively been applied in a concrete infrastructure due to their primary benefit of reducing dead load in structures. Many types of lightweight cementitious materials—such as lightweight aggregate concrete (LWAC), pervious concrete, and aerated concrete—have been developed for various purposes. LWAC typically comprises cement, lightweight aggregate (LWA), water, and mineral and chemical admixtures. In general, the ranges of density and compressive strength of LWAC are 1460–1910 kg/m^3^ and 36.5–60.0 MPa, respectively [1,2,3,4]. Because of its low density and moderate strength level for structural purposes, LWAC has successfully been applied for bridge components [5,6]. Pervious concrete, also called porous concrete, has a macro-porous structure using gap-graded coarse aggregates without fine aggregates in the mixture [7,8]. In pervious concrete, the coarse aggregate particles are typically coated in a thick layer of cementitious paste, resulting in a formation of highly interconnected macro-pore network [9]. To enhance the pore ratio of pervious concrete, natural and artificial fibers can be used despite the reduction of strength and freeze-thaw resistance [10]. Pervious concrete has widely been used for pavements, vegetation concrete beds, and noise absorbing concrete [11,12]. Its ranges of porosity and compressive strength are typically 15–25% and 22–39 MPa, respectively [12]. Aerated concrete has a large number of uniformly distributed small pores that can be generated by adding metallic powder (e.g., aluminum and zinc) and/or a foaming agent (e.g., glue resin and saponin) [13,14,15]. Because of its good heat conservation and low density (300–1800 kg/m^3^), aerated concrete can be used as a high-efficiency heat-insulating material in roofs, walls, and floors—as a form of block and panel—despite its relatively low mechanical properties [16,17,18]. As various properties of lightweight cementitious materials, such as density, compressive strength, elastic modulus, water drainage, heat conservation, and noise absorption, are highly related to their pore characteristics, various methods for analyzing the pore structures have been introduced [19,20,21,22,23,24].

Even though many testing methods have been utilized for analyzing the pore structures of cementitious materials, a suitable testing method should be carefully selected by considering the properties of testing specimens and the limitations of techniques. The most convenient and widely used method is the water absorption test, which measures the difference between the saturated and oven-dried masses of a testing specimen [25,26]. This test provides the total volume of water permeable pores by filling internal pores with water. Similarly, a gas pycnometer can determine the porosity and density of a testing sample using nearly ideal gas (e.g., helium or nitrogen) [27,28]. Although this method limits the size of a testing specimen, due to its small chamber size, the volume of the solid phase can be measured more accurately by detecting the pressure change caused by the gas displacement than with the water absorption test [28]. However, neither the water saturation nor the gas pycnometer method can provide pore characteristics, such as pore size and their spatial distribution. The mercury intrusion porosimetry (MIP) method can evaluate various pore properties (porosity and pore size) by measuring the volume of intruded non-wetting liquid (e.g., mercury) at different pressures [29,30]. However, the MIP method also has some drawbacks regarding the accessibility issue of mercury in the pore throat, resulting in a true pore diameter measuring error [31,32].

As a way to make up for the weakness of the aforementioned methods, the image processing and X-ray computed tomography (X-ray CT) can be applied for analyzing the characteristics of pores based on two-dimensional (2D) and three-dimensional (3D) visual images of specimens, respectively. Using a digital camera and/or a microscope, the image processing technique was conducted to investigate the size of pores, its spatial distributions, and 2D pore ratios [33,34]. This image processing approach generally consists of a collection of high-resolution images, a conversion of an RGB image into a grayscale image, a determination of a threshold, and image binarization [35]. With the use of a high-quality binary image, the detection and quantification of pores can be carried out well. However, the difference between the porosity and 2D pore ratio obtained from the image processing should be considered. Similarly, X-ray computed tomography (X-ray CT) is also available for pore structure analysis based on a 3D tomographic image of a sample [36,37]. Because X-ray CT analysis scans the entire testing specimen and virtually reconstructs actual pore structures, this method can provide information about the internal pore microstructure, 3D pore ratio, pore size, and their spatial distribution [38,39]. Nevertheless, this test is rather expensive for the analysis and requires time-consuming post processing due to a large scanning data that needs to be analyzed. Despite their drawbacks, these testing methods are beneficial for investigating the characteristics of the pore structure based on the images. However, as a comparative analysis of different methods—conventional method, image processing, and X-ray CT—has not been fully studied yet, further research is still needed to select a suitable testing method for highly porous cementitious materials.

In this study, a comparative analysis that uses three different methods—conventional water absorption test, microscopic image processing and X-ray CT analysis—was conducted for investigating highly porous structures of cementitious materials. All 12 porous cementitious material series were prepared using different amounts of pore generation materials of aluminum powder and natural fibers. To analyze the pore structures, a conventional water absorption test was first conducted in order to obtain their water permeable porosities. In addition, their bulk specific gravities were measured. The microscopic image processing method, which uses a local thresholding algorithm, was adopted to characterize the pores in terms of the 2D pore ratio, the pore size, and the number of pores in a cross-section. X-ray CT analysis was also used to provide information about the pore structures—the 3D pore ratio, the pore size, the number of pores, and their spatial distribution—on the basis of a 3D tomographic image.

## 2. Materials and Mix Proportions

To fabricate highly porous cementitious materials, aluminum powder (ECKART, Hartenstein, Germany) and natural fibers (Soo Industry, Gyeongju, Republic of Korea) were added to the mixtures. The water-to-binder ratio (w/b) of cementitious materials was fixed at 0.3. The binders used in the cementitious materials were CEM I 42.5 R ordinary Portland cement (OPC) (Ssangyong Cement, Seoul, Republic of Korea) and silica fume (Elkem, Oslo, Norway). The specific gravity and Blaine fineness of the OPC were 3160 kg/m^3^ and 330 m^2^/kg, respectively. Silica fume had a specific gravity of 2270 kg/m^3^ and replaced 10 wt% of the OPC in order to control viscosity, as well as prevent segregation. The length of the kenaf natural fibers was approximately 1 mm. These fibers consisted of cellulose of 45–57 wt%, hemicellulose of 22 wt%, and lignin of 8–13 wt%, having a tensile strength and Young’s modulus of 930 MPa and 53 GPa, respectively. Its specific gravity was 1800 kg/m^3^, which was determined using a gas pycnometer (Micromeritics AccuPyc 1330, Norcross, GA, USA). The amount of natural fibers used in the cementitious materials was controlled up to 5.0 wt% of mixtures. Aluminum powder was a flake type with a 99.7% purity. Its specific gravity and covering capacity were 2700 kg/m^3^ and 1100–1450 m^2^/kg, respectively. The size of the aluminum powder particles ranged from 12 to 86 µm, with a mean size of 34 µm, as measured using laser diffraction (Sympatec Helos, Clausthal-Zellerfeld, Germany). The amount of aluminum powder was controlled up to 0.1 wt% of binders. A polycarboxylate-based superplasticizer (MasterGlenium SKY 8808, Ludwigshafen, Germany) was added in order to enhance the workability, as well as the homogeneity of mixtures. The dosage of superplasticizer was fixed at 2.0 wt% of binders. As provided in Table 1, a total of 12 mixtures were fabricated following the mixing process. OPC, silica fume, and aluminum powder (if any) were first dry mixed. Then, superplasticizer in liquid form was added to the mixing water. Natural fibers (if any) were added to the superplasticizer-concentrated water. Subsequently, all constituents were mixed in a planetary mixer for 10 min. The fresh mixtures were then cast into a 50 mm cube mold in order to create the specimens to be used for analyzing pore structure and density. The samples were cured in a water chamber for 28 days at a temperature of 23 °C.

## 3. Test Methods for Analyzing the Pore Structures

### 3.1. Water Absorption Test

A water absorption test was conducted to determine the water permeable porosity of cementitious materials following ASTM (American Society for Testing and Materials) C 642. First, the oven-dried mass *(M_oven_)* of the testing specimen was measured, which was previously dried in an oven at a temperature of 110 ± 5 °C for not less than 24 h. Afterward, the saturated surface-dried mass (*M_SSD_*) was determined by measuring the mass of the specimen that was immersed in water for 7 days to saturate porous structure of samples. Subsequently, the water absorption capacity (*P_wat_*) was calculated as [25]

(1) $$P_{wat}[\%]=\left(M_{SSD}-M_{oven}\right)/M_{oven}\times 100$$

The water absorption capacity stands for the water permeable porosity of the testing sample. Considering very low density of testing specimens, their dry bulk densities were measured following ASTM C 1693 [40]. The dry bulk density of the specimen was equal to the oven-dried mass divided by the volume of the sample (i.e., 125,000 mm^3^ in this study). Both the water permeable porosity and the dry bulk density were determined by the average value of 6 specimens in the form of 50 mm cubes.

### 3.2. Microscopic Image Processing

**Figure 1:**
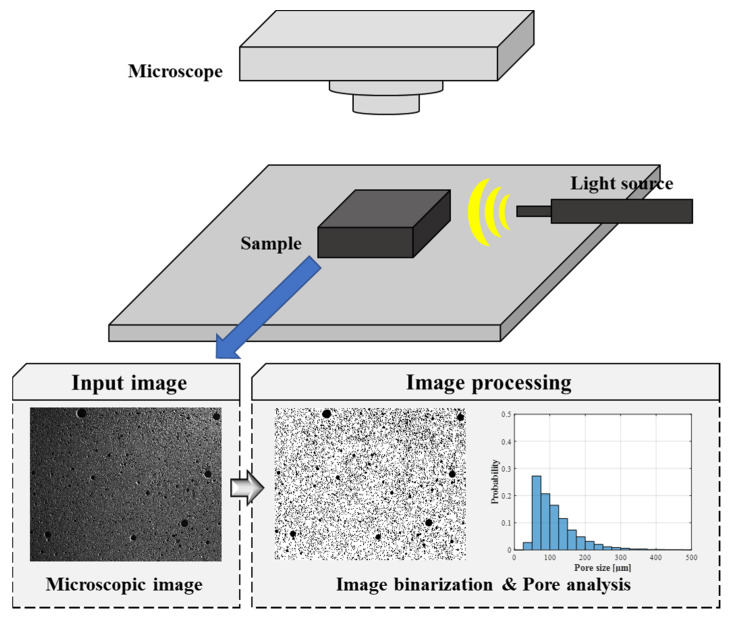
Schematic diagram for the microstructural image processing method.

The aims of microscopic image processing are to distinguish the cementitious matrix and pores and to provide information about the pore size, the pore ratio, and the number of pores in a tested cross-section. This method was performed in three parts: acquisition of microscopic image, image binarization, and characterization of pores (see Figure 1). The cross-sections of specimens were prepared using a cutter (Allied High Tech Products Inc PowerCut 10x, Compton, CA, USA) at the height of approximately 25 mm from the bottom. The cross-sectional images of these samples were collected using an optical microscope (ZEISS Axio Zoom V16 and ZEISS PlanApo Z 0.5x, Oberkochen, Germany). It should be noted that no observable cracks were identified in microscopic images, which indicates that the cutting process did not affect the characterization of pore in cementitious materials. The size of the observed area via the optical microscope was a 28.64 × 35.70 mm^2^ rectangular region with a 12.97 µm/pixel resolution. Through the use of a lateral light source, a shadow was created in the pore, which enabled image processing to classify both the solid phase and the pores well. Subsequently, the image obtained was converted into grayscale, with a brightness range from 0 to 255. During the binarization process, the brightness of each pixel in an image was compared to a threshold. As provided in Equation (2), the brightness of pixels that was less than the threshold was designated as zero, while those having higher values became one—resulting in a binary image (*B_img_*).

(2) $$B_{img}(i,j)=0\;\mathrm{if}\;M_{img}(i,j)<t,\;\mathrm{otherwise}\;B_{img}(i,j)=1,\;\mathrm{where}\;1\;\leq \;i\leq \;a\;\mathrm{and}\;1\;\leq \;j\;\leq \;b,$$

where *M_img_(i,j)* is the pixel intensity of grayscale image, *t* is the brightness threshold, and *a* and *b* are the number of pixels along the width and height. Lastly, using the binary image, the 2D pore ratio—the ratio of the number of pixels for pores to the total number of pixels in the image—and pore size—equivalent circular area diameter of irregularly shaped pores—were numerically calculated. Because these values are influenced by the quality of binary image, a suitable threshold value was carefully determined in order to obtain a reliable classification of pores and cementitious matrix.

The threshold value can be determined using the global and local thresholding methods. The global method (e.g., the Otsu method) uses one threshold value for the binarization of the entire image [41,42]. Although this method is computationally simple and quick, the threshold value depends on the operator or histogram of pixel intensity. Consequently, a poor binarization result can be obtained if the image had a noisy or complex background [42]. On the other hand, the local thresholding method uses the brightness of detected pixels in a neighborhood around a pixel (i.e., window) for the calculation of the unique local threshold value. Although this local method demands more computational cost, the local characteristics of pixels in varying background images can be identified well, including illuminated or degraded images [43,44].

Because non-uniform illumination microscopic images—caused by the lateral light source (see Figure 1)—were used in this study, a local thresholding method was selected. More specifically, Sauvola’s image binarization algorithm—used for document analysis and concrete crack identification—was adapted, as provided in Equation (3) [44,45].

(3) $$T_{Sauvola}=m\cdot (1-k\times (1-s/R))$$

where *T_Sauvola_* is threshold value, *k* is the sensitivity, *R* is the normalized standard deviation, and *m* and *s* are the average and standard deviation of the brightness value of a pixel in a selected local area (window), respectively. In this study, the size of the window and the sensitivity used for the computation were user-defined values. The sensitivity value was fixed at 0.5, which showed a good result in the previous study [44]. Although this sensitivity caused several false detections in PCM1-1 and PCM1-2, due to their rough surfaces, the errors were very small and acceptable because of their lower porosity. The optimal window size was determined to be in the range of 10–300 pixels when comparing changes of the total number of detected pixels for the pores. This value could reach the plateau value, if the classification of the cementitious matrix and the pores were performed well.

### 3.3. X-ray CT Analysis

X-ray CT has strong advantages for analyzing the internal structure of cementitious materials, providing information about internal defects (i.e., pores), as well as visualized 3D anatomical images [46,47,48]. 3D images from X-ray CT can be obtained through two steps: image acquisition and reconstruction. First, a microfocus X-ray beam passes through a sample and 2D X-ray projection images are acquired. The sample that is mounted on the stage is rotated in the 180°–360° range and additional 2D projections are obtained, typically 500–1000 images. Afterward, these projection images are converted into a complete 3D tomographic image of the sample, through a reconstruction process using a back-projection algorithm. Further analysis of the 3D image provides the volumetric information of the solid phase and internal defects on the basis of 3D volumetric elements (voxels). In the tomographic image, a denser phase has a high X-ray attenuation and is displayed as a bright color, whereas a low-density phase—consisting of air or gas—would appear as quite dark due to little X-ray attenuation. Typically, no special pre-processing is required for sample preparation. In a porosity analysis using a software of VG Studio and myVGL 3.0 (Volume Graphics, Heidelberg, Germany), the defect detection algorithm compared the potential defect with its local neighborhood. If the appearance of a potential defect was very similar to the surrounding structure, its probability was reduced, which started from zero. Based on preliminary tests, probability threshold of 0.5 was selected to classify pores and cementitious matrix. The value for minimum pore size was no less than 8 voxels edge lengths (2 × 2 × 2 voxels).

**Figure 2:**
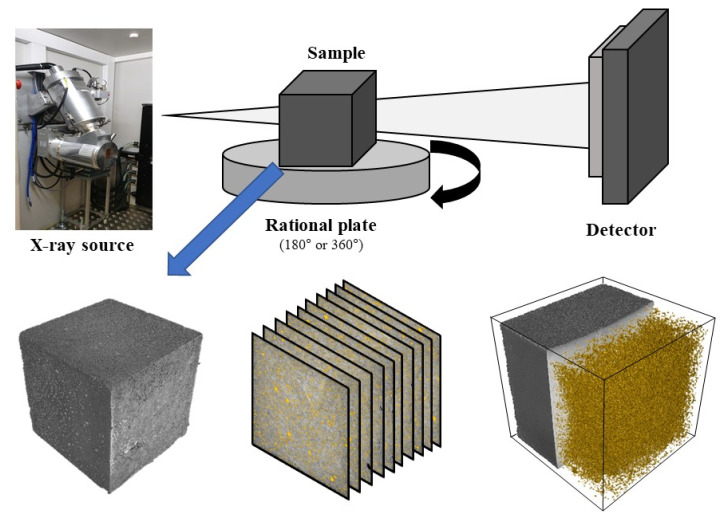
Schematic diagram of the X-ray CT analysis.

In this study, X-ray CT (Nikon Metrology XT H 320, Tokyo, Japan) was used for characterizing the porous internal structures of cementitious materials as shown in Figure 2. A 50 mm cube specimen was mounted on the 360° rotational stage and the 3D image was acquired at a 230 kV accelerating voltage and a 300 µA current. A total of 1140 pictures were obtained from each X-ray CT analysis, taking 354 ms for each image projection. These 2D images were converted into 3D tomographic images consisting of voxels with an edge length of 48 µm. A total of four cementitious samples were examined through the X-ray CT test. Their porosity and pore size results were compared to the water absorption capacity and microscopic image processing-based analysis. Furthermore, the volume of open and closed pores in cementitious materials can be analyzed. The open and closed pores stand for a cavity or channel with access to an external surface and a pore not connected to the surface, respectively. Because of these advantages of X-ray CT, this test could be applied for various purposes, such as internal structure analysis of asphalt concrete [49] and permeability of oil-well cement [50].

## 4. Experimental Results and Discussion

### 4.1. Density and Water Absorption Capacity

**Figure 3:**
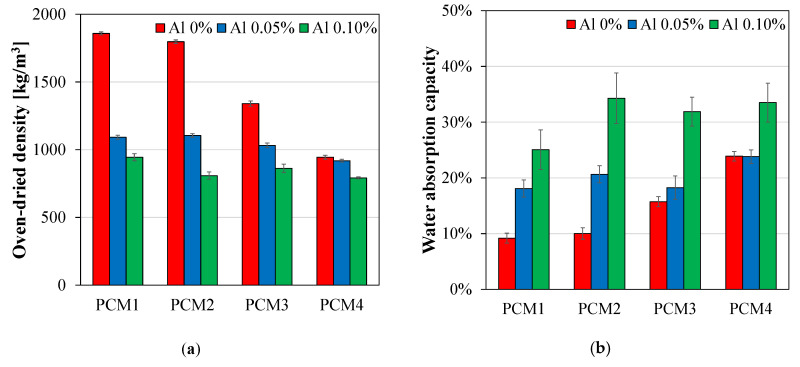
(**a**) Oven-dried density and (**b**) water absorption capacity of the tested porous cementitious materials.

It is known that porous cementitious materials generally have a low density with a high-water absorption capacity [37], which was also identified in this study. As shown in Figure 3, the specimens—which incorporated aluminum powder and natural fibers—showed a decrease in the oven-dried density and an increase in the water absorption capacity. In the PCM1 series, which contained 0–0.1 wt% of aluminum powder, the densities were drastically decreased from 1859 kg/m^3^ to 944 kg/m^3^. The PCM2-1, PCM3-1, and PCM4-1 samples, which incorporated 1.0–5.0 wt% of natural fibers, also showed a decrease in densities from 1797 kg/m^3^ to 944 kg/m^3^. The synergistic effect of these two materials was also observed. For example, the PCM4-3 sample, which contained 0.1 wt% of aluminum powder and 5.0 wt% of natural fibers, showed the lowest density of 792 kg/m^3^. The decrease of the oven-dried density for cementitious materials was highly related to the reaction of aluminum powder and the dispersion of natural fibers. More specifically, the chemical reaction of aluminum powder with calcium hydroxide and water generated air bubbles (hydrogen gas) in the fresh state, resulting in the formation of porous structures [51,52]. In case of natural fibers, the dispersion of fibers during the mixing process can create entrapped air pores in the matrix phase [10,37]. Therefore, the use of these pore generation materials was effective for making highly porous cementitious materials that have low densities.

It was also revealed that the water absorption capacities of the samples were inversely proportional to their densities, as shown in Figure 3b. In the PCM1 series, which incorporated 0–0.1 wt% of aluminum powder, the water permeable porosity significantly increased from 9.2% to 25.1%. The PCM2-1, PCM3-1, and PCM4-1 samples, which contained 1.0–5.0 wt% of natural fibers, also showed an increase in the porosity from 10.0% to 23.9%. The combination of aluminum powder and natural fibers significantly increased the porosity of the samples. Based on this experimental work, the oven-dried density and water absorption capacity of samples were used as references for a comparison with the results obtained from microscopic image processing and X-ray CT analysis.

### 4.2. Microscopic Image Processing Analysis

Following Section 3.2, microscopic image processing was conducted in order to characterize the porous structures of cementitious materials in terms of their 2D pore ratio, the pore size and the number of pores. Because of the non-uniform illumination characteristics of microscopic images, the local thresholding method (Sauvola’s method) was applied for the image binarization. In this section, the optimal window size for the local method was identified to obtain a high-quality binary image. Using this binary image, the characteristics of pores were numerically calculated.

#### 4.2.1. Image Binarization Using the Local Thresholding Method

**Figure 4:**
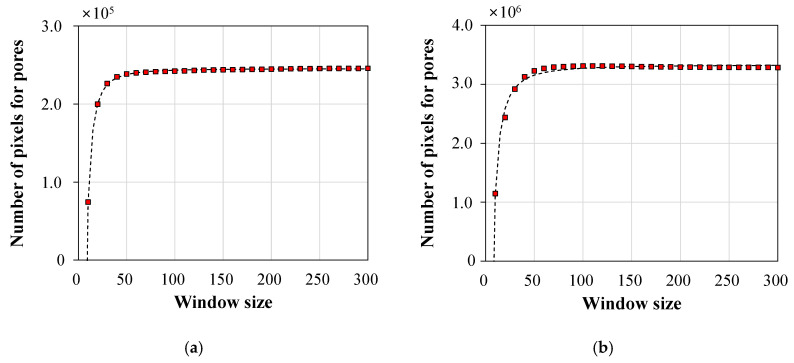
The relationship of the number of pixels designated as pores with the window size: (**a**) PCM1-1 and (**b**) PCM3-3.

**Figure 5:**
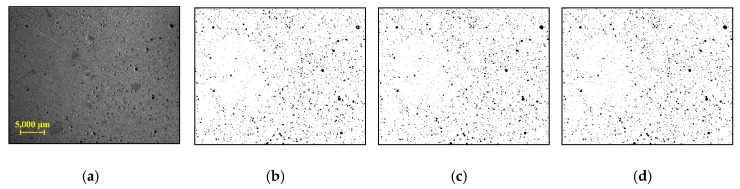
PCM1-1: (**a**) microscopic image; (**b**–**d**) binary images using 50 × 50, 100 × 100, and 200 × 200 windows, respectively.

**Figure 6:**
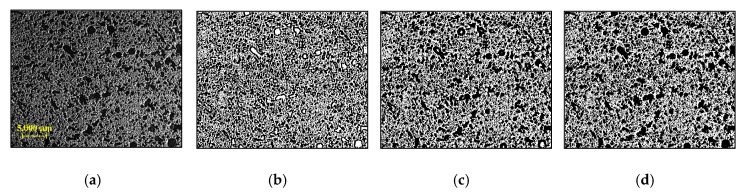
PCM3-3: (**a**) microscopic image; (**b**–**d**) binary images using 50 × 50, 100 × 100, and 200 × 200 windows, respectively.

In the analysis of microscopic image processing, a suitable user-defined parameter—window size—for the local method was first determined to obtain high quality binary image. In Figure 4, Figure 5 and Figure 6, the results obtained from representative samples—the less porous PCM1-1 and the highly porous PCM3-3—are provided in order to show the effects of different window sizes on image binarization. First, the total number of pixels for pores in the cross-section was counted using different window sizes of 10–300 pixels (see Figure 4). As the size of the window increased, the number of pixels for pores also rapidly increased, showing a plateau convergence at a 50-pixel window for PCM1-1 and PCM3-3. This result indicated that the classification of pores can be achieved well using a window size at this convergence value. Therefore, the window sizes of 50, 100, and 200 pixels were selected to evaluate the quality of binary images. As shown in Figure 5, the binary images for the PCM1-1 sample did not show a big difference, regardless of the window size. On the contrary, the large window size of 200 × 200 pixels showed good binarization performance for the highly porous PCM3-3 sample (see Figure 6). Small windows were not enough to cover several large-sized pores in the PCM3-3 sample, resulting in a false binarization for the inside of some large pores. Hence, the 200 × 200 window size was selected for Sauvola’s algorithm in microscopic image processing.

#### 4.2.2. Characteristics of Pores

Using binary images, the characteristics of pores can be identified in terms of the 2D pore ratio, the pore size, and the number of pores, as provided in Table 2. In the PCM1 series, the 2D pore ratio increased from 4.0% to 43.9% with aluminum powder of 0–0.1 wt%. The PCM2-1, PCM3-1, and PCM4-1 samples, containing natural fibers of 1.0–5.0 wt%, showed a 2D pore ratio of 6.7–26.5%. Here, PCM3-3 showed the highest 2D pore ratio of 54.2%, using the combination of aluminum powder of 0.1 wt% and natural fibers of 3.0 wt%.

**Figure 7:**
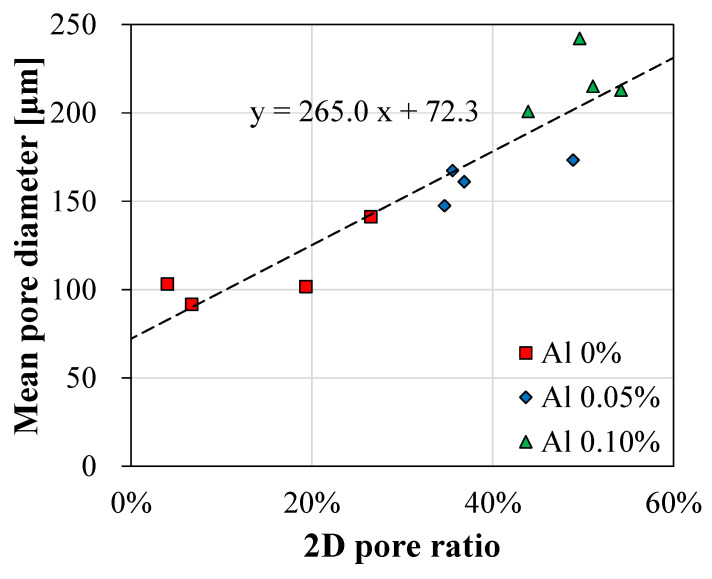
The relationship of the 2D pore ratio with the mean pore diameter obtained using the local thresholding image processing method.

In the analysis of the mean pore size, it was revealed that the mean pore size was increased with a dosage of 0–0.1 wt% aluminum powder. The mean pore size of the PCM1 series ranged from 103.2 µm for PCM1-1 to 200.9 µm for PCM1-3 because of pore generation resulting from the aluminum powder reaction. In addition, the increase in the mean pore size was observed for PCM2-1, PCM3-1, and PCM4-1 as the amount of natural fibers increased. The relationship between the 2D pore ratio and the mean pore diameter was linearly proportional for all 12 samples, as shown in Figure 7.

The number of pores did not proportionally increase as the amount of aluminum powder and natural fibers increased. This is because the number of pores for a given observed area was decreased as the size of pores increased for highly porous cementitious materials. Meanwhile, PCM1-1, PCM2-1, and PCM3-1 samples having low 2D pore ratio and relatively small mean pore size showed an increase of the number of pores as the amount of natural fiber increased.

### 4.3. X-ray CT Analysis

**Figure 8:**
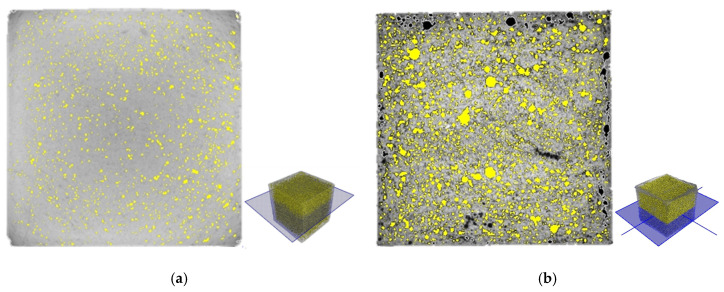
Cross-sectional images of (**a**) PCM1-1 and (**b**) PCM3-3 from the X-ray CT analysis.

X-ray CT analysis provided cross-sectional and 3D tomographic images of the porous cementitious materials, as well as information about the 3D pore ratio, the pore size, and the number of pores. In order to show the effects of aluminum powder and natural fibers, four samples—PCM1-1, PCM 1-3, PCM3-1, and PCM3-3—were selected for the X-ray CT analysis. Figure 8 shows the cross-sectional images of PCM1-1 and PCM3-3. As discussed in Section 4.2, the PCM1-1 control sample had a less porous structure in comparison to the PCM3-3, which incorporated aluminum powder of 0.1 wt% and natural fiber of 3.0 wt%. In Figure 8, the colors of open and closed pores are designated as black and yellow, respectively.

Based on the volumes of the solid phase and the closed pores, the volume of the open pores, as well as the porosity of the samples, can be calculated. It was assumed that the porous concrete specimen consisted of the cement matrix phase, as well as open and closed pores. Then, the volume of the open pores was calculated by subtracting the volumes of the solid phase and the closed pores from the sample volume. The volume of the 50 mm cube specimen was assumed to be 125,000 mm^3^. The volumes of the solid phase and the open and closed pores were provided in Table 3. The total 3D pore ratios of the PCM1-1, PCM1-3, PCM3-1, and PCM3-3 samples were 8.4%, 21.3%, 9.3%, and 23.3%, respectively. The difference between the water permeable porosity, the 2D pore ratio, and the 3D pore ratio is discussed in Section 4.4. Their mean pore sizes were 347.2 μm, 302.2 μm, 335.7 μm, and 360.4 μm, respectively, where the high values were caused by the limited voxel resolution of X-ray CT. The total numbers of closed pores for PCM1-1, PCM1-3, PCM3-1, and PCM3-3 were 194,425, 212,650, 133,471, and 272,721, respectively.

**Figure 9:**
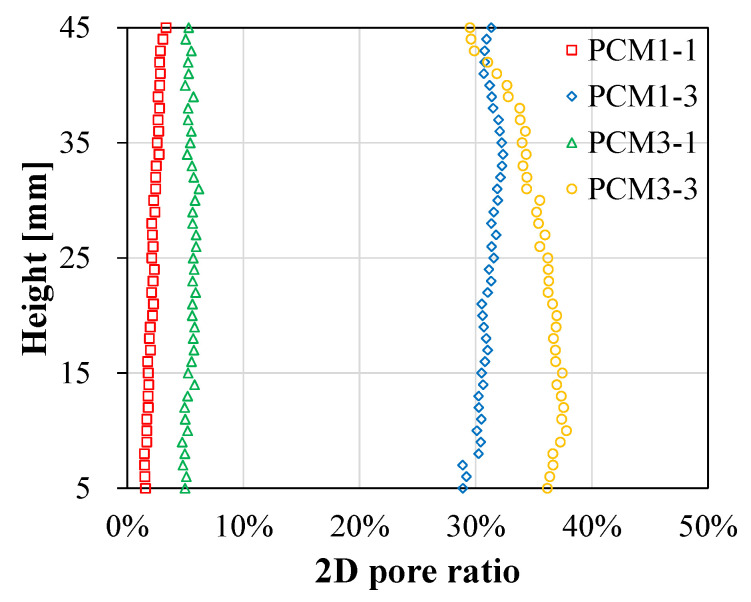
Distribution of the 2D pore ratios obtained through X-ray CT analysis along the height direction.

X-ray CT analysis can be applied for evaluating the distribution of the pore ratio based on the image processing technique [53]. Here, the cross-sectional images obtained from X-ray CT were collected along the height direction at 1 mm intervals. As shown in Figure 9, the 2D pore ratios of PCM1-1, PCM1-3, PCM3-1, and PCM3-3 were 1.5–3.3%, 4.7–6.2%, 28.9–32.4%, and 29.5–37.8%, respectively. This result revealed that the PCM1-1, PCM1-3, and PCM3-1 samples had homogeneous distributions of the pore ratio along the height direction. In case of the PCM3-3 sample, which incorporated both aluminum powder and natural fibers, the pore ratio was inconsistent along the height. This is because some natural fibers agglomerated on the top surface, resulting in the formation of a fiber ball, as well as in the reduction of the pore ratio in the matrix [54].

The difference between the average 2D pore ratio and 3D pore ratio obtained from the X-ray CT were identified. The average 2D pore ratios were 2.2%, 31.0%, 5.4%, and 35.2% for PCM1-1, PCM1-3, PCM3-1, and PCM3-3, respectively. These results were different from their 3D pore ratios of 8.4%, 21.3%, 9.3%, and 23.3%, respectively. This indicated that the less porous sample had a relatively low 2D pore ratio, while the highly porous one had a high 2D pore ratio. This was caused by the different calculation for area of circle and volume of sphere. A detailed discussion is provided in Section 4.4.

As provided in this experimental result, X-ray CT analysis was beneficial for analyzing various characteristics of pores, such as open and closed pores, pore distribution in 3D space, and 3D tomographic images. Furthermore, the homogeneous pore distribution can be evaluated using the cross-sectional image processing. These results are highly related to the unique properties of porous cementitious materials, such as heat insulation, sound absorption, and water drainage. Consequently, more diverse applications of the X-ray CT method are expected in the field of construction materials.

### 4.4. Comparative Analysis

**Figure 10:**
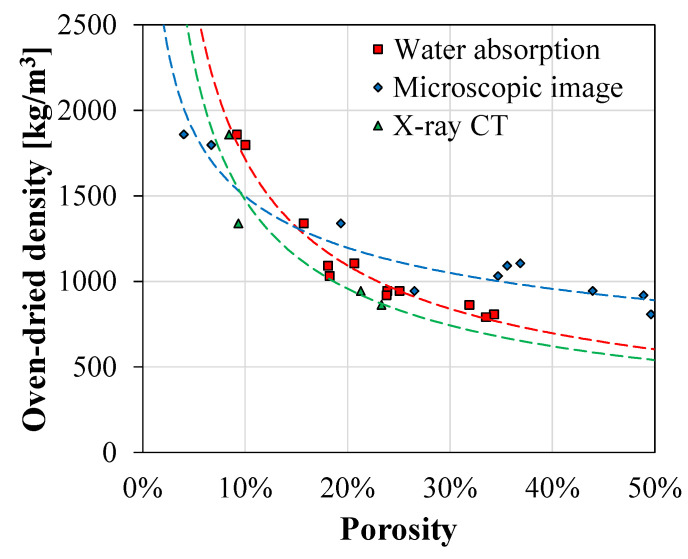
The relationship of the oven-dried density with the porosities obtained using the water absorption test, microscopic image processing, and X-ray CT analysis.

In this section, the characteristics of the porous structures obtained from the water absorption test, microscopic image processing, and X-ray CT analysis were compared. First, the relationships of the oven-dried densities and porosities determined by these different testing methods were investigated, as shown in Figure 10. The regression curves for the water permeable porosity and the 3D pore ratio showed similar trends. This is because both methods determined the porosities of the specimens by considering all 3D pores in them. Furthermore, testing four specimens for X-ray CT analysis—covering a wide range of porosities and densities—was adequate to estimate the trend for all 12 samples. It should be noted that the difference of the 3D pore ratio and the water permeable porosity for the highly porous samples might be attributed by the classification errors of the cementitious matrix and the pores due to the limited voxel resolution of X-ray CT.

On the other hand, the results from microscopic image processing showed a relatively low porosity for the high-density sample and a high porosity for the low density one. The low 2D pore ratio might be attributed to small-sized pores, which were quite bright and designated as matrix due to shallow depths. The high 2D pore ratio obtained from microscopic image processing was caused by different calculations. Let us assume that a pore with a 1 mm radius was located inside a cube that is 2 mm in length. The 3D pore ratio would be 0.52 and the 2D pore ratio, at the center, would be 0.79. Because the cross-sections of the highly porous samples were almost fully filled with pores, the 2D pore ratio was always higher than the water permeable porosity and the 3D pore ratio obtained using X-ray CT. Furthermore, closed pores could represent a portion of the pores in a high-resolution microscopic image—and they cannot be saturated by water. For these reasons, there was a difference between the results obtained from the 3D-based testing methods—water absorption test and X-ray CT—and the 2D-based image processing. Hence, considering the different characteristics of each testing method, their complementary uses are recommended for analyzing the porous structures of cementitious materials well.

## 5. Conclusions

This experimental study conducts a comparative analysis for characterizing the pores in cementitious materials by adopting microscopic image processing and X-ray CT. All 12 porous cementitious samples were fabricated using various dosages of aluminum powder and natural fibers. The porous structures were evaluated in terms of the pore size, the number of pores, the spatial distribution of pores along the height, and open and closed pores. The key observations and findings of this study are summarized as follows:

1. In microscopic image processing, the local thresholding method was adopted by considering non-uniform illumination images caused by a lateral light source. As a preliminary study, user-defined parameters of window size and sensitivity were carefully selected as 200 × 200 and 0.5, respectively. Consequently, microscopic image processing was successfully performed and various characteristics of pores were provided using high quality binary images. Furthermore, the linear relationship between the 2D pore ratio and the mean pore diameter was identified.
2. X-ray CT analysis was conducted for the representative samples with a wide range of porosities. This 3D tomographic image-based analysis provided various unique characteristics of pores, such as open and closed pores and the distribution of pores in the 3D space. However, a high-resolution 3D tomographic image is required in order to obtain a more accurate analysis on the porous structures.
3. To compare the properties of porosity obtained using different testing methods, the relationship of porosity with oven-dried density was investigated. The regression curves obtained for the water permeable porosity and the 3D pore ratio using X-ray CT showed similar trends. On the other hand, the results obtained using microscopic image processing provided a low 2D pore ratio for dense materials and a high 2D pore ratio for porous materials due to both the calculations used and the portion of closed pores that the samples contained.

As technology advances, it is expected that a high-resolution 3D tomographic image from X-ray CT would provide a more accurate analysis for the characteristics of porous cementitious materials. Furthermore, it might also be beneficial for investigating the relationships of porous cementitious materials with their unique properties, such as heat conservation, noise absorption, and water drainage.

## Figures and Tables

**Table 1:** Mix proportions of porous cementitious materials.

Label	Mix Proportion (g)						
	w/b^1^	Water	Cement	Silica Fume	NF^2^	AP^3^	SP^4^
PCM1-1	0.3	660	2000	200	0	0 1.1 2.2	44.0
PCM1-2							
PCM1-3							
PCM2-1					28.6	0 1.1 2.2	
PCM2-2							
PCM2-3							
PCM3-1					85.8	0 1.1 2.2	
PCM3-2							
PCM3-3							
PCM4-1					143.0	0 1.1 2.2	
PCM4-2							
PCM4-3							

w/b^1^: water-to-binder ratio,NF^2^: Natural fibers, AP^3^: Aluminum powder, SP^4^: Superplasticizer.

**Table 2:** Microscopic image processing-based pore analysis using the local method.

Measurement	Label	Aluminum Powder		
		0%	0.05%	0.10%
2D Pore Ratio	PCM1	4.0%	35.6%	43.9%
	PCM2 (NF 1%)	6.7%	36.9%	49.6%
	PCM3 (NF 3%)	19.3%	34.7%	54.2%
	PCM4 (NF 5%)	26.5%	48.9%	51.1%
Mean Pore Size (µm)	PCM1	103.2	167.3	200.9
	PCM2 (NF 1%)	91.7	161.0	242.1
	PCM3 (NF 3%)	101.7	147.4	212.9
	PCM4 (NF 5%)	141.2	173.3	215.0
Total Number of Pores	PCM1	3568	10,317	8397
	PCM2 (NF 1%)	8234	11,669	4914
	PCM3 (NF 3%)	18,421	13,384	5282
	PCM4 (NF 5%)	10,513	7984	6023

**Table 3:** X-ray CT-based analysis for the porous structures of PCM1-1, PCM1-3, PCM3-1, and PCM3-3.

Label	Volume (mm^3^)			3D Pore Ratio (Open/Closed)	Mean Pore Size (μm)
	Solid Phase	Closed Pore	Open Pore		
**PCM1-1**	114,451	5616	4933	8.4% (3.9%/4.5%)	3472
**PCM1-3**	98,395	21,443	5162	21.3% (4.1%/17.2%)	3022
**PCM3-1**	113,337	7325	4339	9.3% (3.5%/5.9%)	3357
**PCM3-3**	95,860	18,312	9528	23.3% (8.7%/14.6%)	3604

## References

[1] Bogas J.A., Gomes A., Pereira M.F.C. (2012). Self-compacting lightweight concrete produced with expanded clay aggregate. Constr. Build. Mater..

[2] Rossignolo J.A., Agnesini M.V.C., Morais J.A. (2003). Properties of high-performance LWAC for precast structures with Brazilian lightweight aggregates. Cement Concrete Compos..

[3] Andrade L.B., Rocha J.C., Cheriaf M. (2009). Influence of coal bottom ash as fine aggregate on fresh properties of concrete. Constr. Build. Mater..

[4] Choi B.S., Yoon H.S., Moon H.K., Yang K.H. (2018). Environmental impact assessment of lightweight aggregate concrete according to replacement ratio of artificial lightweight fine aggregates. J. Korea Concr. Inst..

[5] Chai Y.H. (2016). Service Performance of Long-Span Lightweight Aggregate Concrete Box-Girder Bridges. J. Perform. Constr. Facil..

[6] Qian Z., Chen L., Jiang C., Luo S. (2011). Performance evaluation of a lightweight epoxy asphalt mixture for bascule bridge pavements. Constr. Build. Mater..

[7] Neithalath N., Sumanasooriya M.S., Deo O. (2010). Characterizing pore volume, sizes, and connectivity in pervious concretes for permeability prediction. Mat. Charact..

[8] Zaetang Y., Wongsa A., Sata V., Chindaprasirt P. (2013). Use of lightweight aggregates in pervious concrete. Constr. Build. Mater..

[9] Chen Y., Wang K., Wang X., Zhou W. (2013). Strength, fracture and fatigue of pervious concrete. Constr. Build. Mater..

[10] Kim H.-H., Kim C.-S., Jeon J.-H., Park C.-G. (2016). Effects on the Physical and Mechanical Properties of Porous Concrete for Plant Growth of Blast Furnace Slag, Natural Jute Fiber, and Styrene Butadiene Latex Using a Dry Mixing Manufacturing Process. Materials.

[11] Kant Sahdeo S., Ransinchung G.D., Rahul K.L., Debbarma S. (2020). Effect of mix proportion on the structural and functional properties of pervious concrete paving mixtures. Constr. Build. Mater..

[12] Chindaprasirt P., Hatanaka S., Chareerat T., Mishima N., Yuasa Y. (2008). Cement paste characteristics and porous concrete properties. Constr. Build. Mater..

[13] Narayanan N., Ramamurthy K. (2000). Structure and properties of aerated concrete: A review. Cem. Concr. Compos..

[14] Benazzouk A., Douzane O., Mezreb K., Quéneudec M. (2006). Physico-mechanical properties of aerated cement composites containing shredded rubber waste. Cem. Concr. Compos..

[15] Yoon H.S., Yang K.H. (2019). Optimum mixture proportioning of autoclave lightweight aerated concrete considering required foaming rate and compressive strength. J. Korea Concr. Inst..

[16] He X., Zheng Z., Yang J., Su Y., Wang T., Strnadel B. (2020). Feasibility of incorporating autoclaved aerated concrete waste for cement replacement in sustainable building materials. J. Cleaner Prod..

[17] Song Y., Li B., Yang E.H., Liu Y., Ding T. (2015). Feasibility study on utilization of municipal solid waste incineration bottom ash as aerating agent for the production of autoclaved aerated concrete. Cem. Concr. Compos..

[18] Liu Y., Leong B.S., Hu Z.T., Yang E.H. (2017). Autoclaved aerated concrete incorporating waste aluminum dust as foaming agent. Constr. Build. Mater..

[19] Stroeven P., Hu J., Koleva D.A. (2010). Concrete porosimetry: Aspects of feasibility, reliability and economy. Cem. Concr. Compos..

[20] Elsharief A., Cohen M.D., Olek J. (2005). Influence of lightweight aggregate on the microstructure and durability of mortar. Cem. Concr. Res..

[21] Chung S.Y., Abd Elrahman M., Kim J.S., Han T.S., Stephan D., Sikora P. (2019). Comparison of lightweight aggregate and foamed concrete with the same density level using image-based characterizations. Constr. Build. Mater..

[22] Ćosić K., Korat L., Ducman V., Netinger I. (2015). Influence of aggregate type and size on properties of pervious concrete. Constr. Build. Mater..

[23] Yoon J.Y., Kim J.H. (2019). Mechanical properties of preplaced lightweight aggregates concrete. Constr. Build. Mater..

[24] Yoon J.Y., Lee J.Y., Kim J.H. (2019). Use of raw-state bottom ash for aggregates in construction materials. J. Mater. Cycles Waste Manag..

[25] ASTM C642-13, Standard Test Method for Density, Absorption, and Voids in Hardened Concrete 2013. https://www.astm.org/Standards/C642.

[26] Cnudde V., Cwirzen A., Masschaele B., Jacobs P.J.S. (2009). Porosity and microstructure characterization of building stones and concretes. Eng. Geol..

[27] Semel F.J., Lados D.A. (2006). Porosity analysis of PM materials by helium pycnometry. Powder Metall..

[28] Yang X., Sun Z., Shui L., Ji Y. (2017). Characterization of the absolute volume change of cement pastes in early-age hydration process based on helium pycnometry. Constr. Build. Mater..

[29] Chen S.J., Li W.G., Ruan C.K., Sagoe-Crentsil K., Duan W.H. (2017). Pore shape analysis using centrifuge driven metal intrusion: Indication on porosimetry equations, hydration and packing. Constr. Build. Mater..

[30] Shah V., Bishnoi S. (2018). Analysis of Pore Structure Characteristics of Carbonated Low-Clinker Cements. Transp. Porous Media..

[31] Zhou J., Ye G., van Breugel K. (2010). Characterization of pore structure in cement-based materials using pressurization-depressurization cycling mercury intrusion porosimetry (PDC-MIP). Cem. Concr. Res..

[32] Diamond S. (2000). Mercury porosimetry: An inappropriate method for the measurement of pore size distributions in cement-based materials. Cem. Concr. Res..

[33] Chung S.Y., Sikora P., Rucinska T., Stephan D., Abd Elrahman M. (2020). Comparison of the pore size distributions of concretes with different air-entraining admixture dosages using 2D and 3D imaging approaches. Mater. Charact..

[34] Martin W.D., Putman B.J., Kaye N.B. (2013). Using image analysis to measure the porosity distribution of a porous pavement. Constr. Build. Mater..

[35] Choi J., Lee Y., Kim Y.Y., Lee B.Y. (2017). Image-processing technique to detect carbonation regions of concrete sprayed with a phenolphthalein solution. Constr. Build. Mater..

[36] Zhang J., Ma G., Ming R., Cui X., Li L., Xu H. (2018). Numerical study on seepage flow in pervious concrete based on 3D CT imaging. Constr. Build. Mater..

[37] Yoon J., Kim H., Koh T., Pyo S. (2020). Microstructural characteristics of sound absorbable porous cement-based materials by incorporating natural fibers and aluminum powder. Constr. Build. Mater..

[38] Yu F., Sun D., Hu M., Wang J. (2019). Study on the pores characteristics and permeability simulation of pervious concrete based on 2D/3D CT images. Constr. Build. Mater..

[39] Zhou H., Li H., Abdelhady A., Liang X., Wang H., Yang B. (2019). Experimental investigation on the effect of pore characteristics on clogging risk of pervious concrete based on CT scanning. Constr. Build. Mater..

[40] ASTM C1693-11, Standard Specification for Autoclaved Aerated Concrete 2017. https://www.astm.org/Standards/C1693.htm.

[41] Wong H.S., Head M.K., Buenfeld N.R. (2006). Pore segmentation of cement-based materials from backscattered electron images. Cem. Concr. Res..

[42] Otsu N. (1979). Threshold selection method from gray-level histograms. IEEE Trans. Syst. Man Cybern..

[43] Gatos B., Pratikakis I., Perantonis S.J. (2006). Adaptive degraded document image binarization. Pattern Recognit..

[44] Sauvola J., Pietikäinen M. (2000). Adaptive document image binarization. Pattern Recognit..

[45] Kim H., Ahn E., Cho S., Shin M., Sim S.H. (2017). Comparative analysis of image binarization methods for crack identification in concrete structures. Cem. Concr. Res..

[46] Manahiloh K.N., Muhunthan B., Kayhanian M., Gebremariam S.Y. (2012). X-ray Computed Tomography and Nondestructive Evaluation of Clogging in Porous Concrete Field Samples. J. Mater. Civ. Eng..

[47] Bernardes E.E., Mantilla Carrasco E.V., Vasconcelos W.L., de Magalhães A.G. (2015). X-ray microtomography (μ-CT) to analyze the pore structure of a Portland cement composite based on the selection of different regions of interest. Constr. Build. Mater..

[48] Hong S., Liu P., Zhang J., Xing F., Dong B. (2019). Visual & quantitative identification of cracking in mortar subjected to loads using X-ray computed tomography method. Cem. Concr. Compos..

[49] Liu T., Zhang X.N., Li Z., Chen Z.Q. (2014). Research on the homogeneity of asphalt pavement quality using X-ray computed tomography (CT) and fractal theory. Constr. Build. Mater..

[50] Golubev I., Karpova Y. (2017). Quality improvement of oil-contaminated wastewater, meant for injection into formation, using two-stage treatment technology. J. Ecol. Eng..

[51] Holt E., Raivio P. (2005). Use of gasification residues in aerated autoclaved concrete. Cem. Concr. Res..

[52] Wang F., Liu Z., Hu S. (2010). Early age volume change of cement asphalt mortar in the presence of aluminum powder. Mater. Struct..

[53] Zhao Y., Wang X., Jiang J., Zhou L. (2019). Characterization of interconnectivity, size distribution and uniformity of air voids in porous asphalt concrete using X-ray CT scanning images. Constr. Build. Mater..

[54] Chakraborty S., Kundu S.P., Roy A., Basak R.K., Adhikari B., Majumder S.B. (2013). Improvement of the mechanical properties of jute fibre reinforced cement mortar: A statistical approach. Constr. Build. Mater..

